# Le cancer différencié de la thyroïde chez l’enfant et l’adolescent: à propos de 22 cas

**DOI:** 10.11604/pamj.2017.28.71.11401

**Published:** 2017-09-25

**Authors:** Said Anajar, Mohammed Tatari, Adil Lakhbal, Reda Abada, Sami Rouadi, Mohammed Roubal, Mohammed Mahtar

**Affiliations:** 1 Service ORL et Chirurgie Cervico-faciale, Hôpital 20 Août, Casablanca, Maroc

**Keywords:** Cancer différencié de la thyroïde, enfant, traitement, pronostic, Differentiated thyroid cancer, child, treatment, prognosis

## Abstract

L’obectif était de mettre en relief les particularités du cancer de la thyroïde chez l’enfant et l’adolescent, et d’évaluer nos résultats par rapport à la littérature internationale a travers une série de cas la plus représentatif au Maroc: 22 cas. C'est une étude rétrospective descriptive des patients atteints de cancer différencié de la thyroïde, hospitalisés au service d’ORL et de Chirurgie Cervico-faciale de L’hopital 20 Août de Casablanca-Maroc, sur la période qui s’étend de Janvier 1995 à Mars 2015. Nous avons recueilli les données relatives à 22 cas, qui répondaient à nos critères d’inclusion. L’âge moyen de nos patients était de 14 ans, avec une sex-ratio 3,4, la plupart de nos patients ont consulté pour un nodule thyroïdien, associé dans 22,7% des cas à une adénopathie cervicale, et dans 9,1% à des signes de compression. L’ensemble des patients ont bénéficié d’une thyroïdectomie totale, suivie d’un curage ganglionnaire dans 31,82%. Le diagnostic de cancer thyroïdien a reposé sur l’examen anatomopathologique de la pièce opératoire, qui a objectivé un carcinome papillaire dans 95,4% des cas, et un carcinome vésiculaire dans 4,5%. Le traitement par l’iode radioactif 131 a été réalisé dans 100% des cas. Par la suite tous nos patients ont été mis sous hormonothérapie thyroïdienne. Une surveillance étroite et régulière a permis de détecter des métastases ganglionnaires chez 3 patients, et les métastases à distance chez 4 patients. Le cancer différencié de la thyroïde de l’enfant et l’adolescent est une entité rare mais agressive, son traitement se base sur la chirurgie, associée à l’irathérapie donnant un pronostic excellent.

## Abstract (en)

This study aimed to highlight the features of thyroid cancer in children and adolescents and to evaluate our results compared to the international literature on the basis of the most representative case series in Morocco: 22 cases. We conducted a retrosective study of patients with differentiated thyroid cancer hospitalized in the otorhinolaryngology and cervicofacial surgery department at the Hôpital 20 Août, Casablanca, Morocco over the period January 1995-March 2015. We collected data on 22 cases that met our inclusion criteria. The average age of our patients was 14 years, with a sex-ratio of 3.4; most of our patients presented with thyroid nodule, associated with cervical lymphadenopathy in 22.7% of cases and with signs of compression in 9.1% of cases. All patients underwent total thyroidectomy, followed by lymph node dissection in 31.82% of cases. The diagnosis of thyroid cancer was based on the anatomo-pathological examination of the surgical specimen, that showed papillary carcinoma in 95.4% of cases and vesicular carcinoma in 4.5% of cases. Radioactive iodine-131 treatment was performed in 100% of cases. Subsequently all patients underwent thyroid hormone therapy. Close and regular monitoring allowed the detection of nodal metastases in 3 patients and of distant metastases in 4 patients. Differentiated thyroid cancer in children and adolescents is rare but aggressive. Treatment is based on surgery associated with iratherapy, with excellent prognosis.

## Introduction

Les cancers différenciés thyroïdiens (CDT) sont des tumeurs malignes épithéliales de souche folliculaire. Chez l’enfant et l’adolescent ce sont des tumeurs rares, en dehors d’une exposition aux radiations comme ce fut le cas en 1986 en Ukraine après la catastrophe de Tchernobyl. ils représente 1,5% de toutes les tumeurs avant 15 ans et 7% des tumeurs de la tête et du cou [1–3]. Le pic maximal de fréquence se trouve entre 7 et 12 ans. Le type histologique papillaire reste majoritaire [1, 3]. Le traitement repose sur la chirurgie, l’iode radioactif puis l’hormonothérapie suppressive de la TSH. Le pronostic est globalement favorable même en cas d’extension initiale importante et même après rechute locale, avec une survie à 20 ans supérieure à 90%. La possibilité de récidive tardive après le traitement initial fait conseiller une surveillance à vie [4]. Le but de notre travail consiste à mettre en relief les particularités du cancer de la thyroïde chez l’enfant et l’adolescent sur les plans épidémiologique, clinique et d’évaluer la réponse au traitement, ainsi que le pronostic a travers une série de cas la plus représentatif au MAROC: 22 cas.

## Méthodes

Nous avons réalisé une étude rétrospective analytique portant sur 22 patients âgés de moins de 18 ans, présentant un CDT, étalée sur 20 ans de janvier 1995 à mars 2015, hospitalisés au service d’oto-rhino-laryngologie et de chirurgie cervico-faciale de l’hôpital 20 Août de Casablanca, nous comparons nos résultats à ceux déjà décrits dans la littérature internationale. Les patients inclus dans notre étude sont ceux, qui ont un carcinome différencié de la thyroïde (CDT) confirmé histologiquement et dont l’âge est inférieur à 18 ans, ont été exclus de notre étude les patients présentant d’autres formes histologiques de cancer de la thyroïde, ou dont l´examen anatomopathologique est non concluant. Les critères que nous étudions sont le sexe, l’âge, l’origine géographique, les examens paracliniques, le traitement réalisé, ainsi que les traitements complémentaires, les résultats anatomo-pathologiques, les suites postopératoires et l’évolution ultérieure.

## Résultats

**Figure 1:**
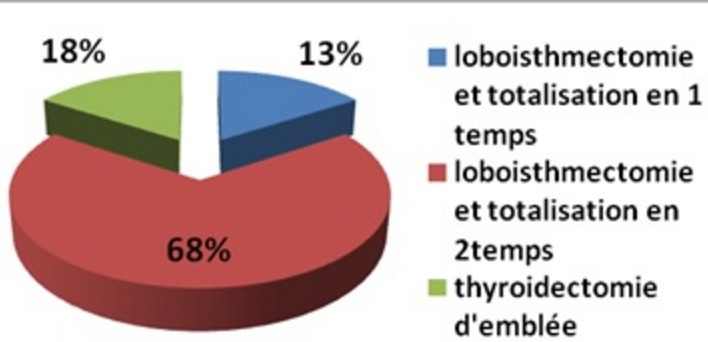
Répartition des malades selon le type chirurgical

**Figure 2:**
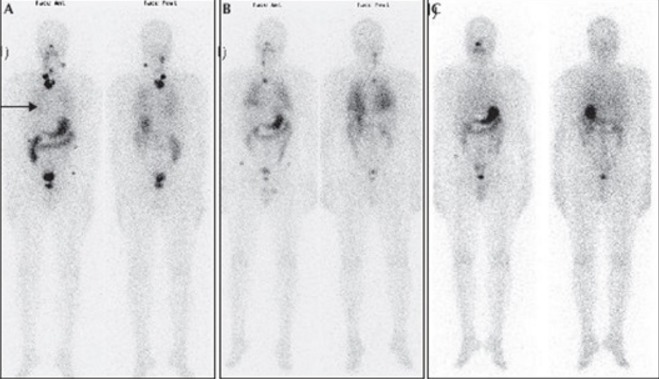
Scintigraphie post-thérapeutique réalisée trois à cinq jours après administration d’iode 131 montrant des métastases pulmonaires bilatérales (flèche noire) associées à des adénopathies cervicales fixantes

Notre série comporte 22 cas, dont 17 filles et 5 garçons soit un sex-ratio F/H de 3,4. Et dont l’âge moyen était de 14 ans sur des extrêmes allant de 6 à 17 ans. La notion d’exposition aux irradiations n’est pas retrouvée, en revanche, on note la notion de goitre familial chez 5 malades soit 22,73%, et 2 cas de carcinomes thyroïdiens soit 9,1%. Le mode révélateur majoritaire était la découverte d’un nodule thyroïdien dans 19 cas (86,4%), associé à: une adénopathie cervicale dans 5 cas (22,7%); des signes de compression à type de dyspnée et dysphagie dans 2 cas (9,1%); des signes cliniques d’hyperthyroïdie dans 2 cas (9,1%). Deux patients ont consulté devant l’apparition d’une adénopathie cervicale isolée et aucun cas de métastase révélatrice n’a été identifié (Tableau 1). Ces signes étaient le plus souvent remarqués par la famille, puis confirmés par une scintigraphie thyroïdienne au technétium 99 métastable (99mTc) ou par une échographie cervicale. Le traitement a consisté en une thyroïdectomie totale chez tous les patients, réparties en une thyroïdectomie d’emblée seulement chez 4 patients, et une totalisation après une loboisthmectomie première chez 18 patients soit lors d’un même temps opératoire après l’examen extemporané de la pièce de loboisthmectomie chez 3 malades ou lors d’un 2^ème^ temps opératoire après l’examen histologique définitif de la pièce de loboisthmectomie chez les 15 autres patients (Figure 1) avec un temps moyen écoulé entre le 1er geste opératoire et la totalisation chirurgicale de 3 mois sur des extrêmes allant de 1 mois à 6 mois. La thyroïdectomie a été associée à un curage ganglionnaire chez 7 patients soit 31,82%. L’examen anatomopathologique a montré un carcinome papillaire chez 21 patients soit 95,45%, associé à une effraction capsulaire dans 9 cas soit 40,91%, des emboles vasculaires chez 7 patients soit 31,82%, la multifocalité et les métastases ganglionnaires chez 6 patients soit 27,27%, et un carcinome vésiculaire chez un seul malade. Les suites postopératoires ont été marquées par une paralysie récurrentielle dans un cas, et une hypoparathyroïdie dans un autre cas, une hypocalcémie passagère a été observée, elle a régressé au bout de 3 jours sous traitement. Le traitement hormonal à base de la LT4 (L-thyroxine), est prescrit aux 22 patients, instauré entre J1 et J3 post-opératoire. Sa posologie est adaptée au poids du patient et au taux de la TSH lors du suivi. Le protocole thérapeutique utilisé, basé sur les données de la classification TNM, conforté parfois par la présence d’un taux élevé de la thyroglobuline (Tg) en défreination a fait que tous les patients avaient bénéficié 3 semaines après arrêt de la L-thyroxine d’une irathérapie complémentaire à forte dose à travers l’administration orale d’une activité thérapeutique d’Iode 131 comprise entre 1,85 et 3,7 GBq (50–100 mCi). Une scintigraphie corps entier est réalisée trois jours après la prise d’iode à dose thérapeutique chez tous les patients objectivant un ou plusieurs résidus thyroïdiens sans foyer suspect sur le reste du corps chez 17 patients, et des foyers cervicaux et pulmonaires ont été révélés chez 4 patients soit 18,18% (Figure 2). Un contrôle biologique par la thyroglobuline validé systématiquement par un dosage des anticorps anti-thyroglobuline a été pratiqué 4 mois après la cure afin d’évaluer l’efficacité du traitement. Au cours du suivi, 4 patients (18,18%) ont présenté des métastases pulmonaires sous forme de miliaire qui n’a été révélée que sur un balayage isotopique diagnostique, et chez qui on a obtenu une carte blanche isotopique au balayage d’efficacité après une cure d’irathérapie. Une récidive ganglionnaire a été notée dans 3 cas (13,64%) en post-chirurgie, dont l’évolution a été favorable après une reprise chirurgicale pour un curage ganglionnaire, comme en témoigne le taux effondré de la thyroglobuline.

**Tableau 1:** Les circonstances de la découverte du cancer

	NOMBRE	POURCENTAGE
Nodule thyroïdien isolé	9	40%
Adénopathie cervicale	2	9%
Nodule thyroïdien +adénopathie cervicale	5	22%
Nodule thyroïdien +signes de compression	2	9%
Nodule thyroïdien + signes cliniques d’hyperthyroïdie	3	13%

## Discussion

Le cancer de la thyroïde chez l’enfant et l’adolescent est très rare, il présente une incidence maximale autour de 15 ans et reste très rare avant 10 ans [5]. Il s’agit d’un cancer papillaire bien différencié dans 90% des cas [6]. On note une nette prédominance féminine avec une fréquence de 69 à 79% selon les séries [7]. Une seule cause connue qui est l’irradiation de la thyroïde, d’autres facteurs pourraient jouer un rôle déterminant comme une prédisposition génétique, la carence en iode stable ou les facteurs hormonaux. Actuellement, les prédispositions génétiques occupent de plus en plus une place prépondérante, si bien que 3 à 5% des patients atteints de cancer de la thyroïde possèdent un membre de la famille atteint aussi d’un cancer de la thyroïde [8]. Dans notre série, aucun antécédent d’exposition aux rayonnements ionisants n’a été signalé, par contre on note 5 cas d’antécédent familiaux de nodule thyroïdien. Les signes révélateurs sont dominés par l’adénopathie cervicale dont l’association avec le nodule thyroïdien reste l’aspect le plus évocateur [9–12]. L’échographie cervical fait partie du bilan minimal afin d’établir un bilan d’extension précis guidant le geste chirurgical. Dans les formes évoluées, la spécificité et la sensibilité de la cytoponction pour affirmer le carcinome thyroïdien peuvent atteindre 100% [13]. En fonction du résultat histologique et selon l’évolution de la maladie, des bilans d’extension pourront être préconisés par le biais d’examens d’imagerie tels qu’un TEP-scan et/ou un scanner cervico-thoraco- abdomino-pelvien et/ou une IRM du rachis et du bassin. Des examens biologiques standards avec des dosages plus spécifiques comme le dosage de la thyroglobuline et des anticorps anti-thyroglobuline sont indiqués. Dans notre série, 19 patients ont bénéficié de ces dosages. Une TEP-TDM a été demandée dans 4 cas, et une radiographie pulmonaire dans 7 cas. La chirurgie complète en un temps est généralement préconisée, associant une thyroïdectomie totale à des curages cervicaux adaptés à l’extension de la maladie [14,15]. Par ailleurs, la morbidité de ces gestes extensifs doit être soulignée , un taux moyen de paralysie récurrentielle et/ou d’hypoparathyroïdie définitive est retrouvé dans la littérature à des taux variant de 5 à 13 % [16,17]. Dans notre série tous les patients ont bénéficié d’une thyroïdectomie totale associée à un curage ganglionnaire chez 7 patients, complétée par une totalisation isotopique chez l’ensemble des malades. Une paralysie récurrentielle a été observée chez un seul malade. L’iode 131 permet d’obtenir un nettoyage des reliquats thyroïdiens et des métastases pulmonaires iodofixantes. Il est d’autant plus efficace que ces métastases sont de plus petites dimensions et non visualisées par les examens morphologiques. La supplémentation orale systématique par L-thyroxine après thyroïdectomie totale sera débutée en postopératoire immédiat ou après le traitement par iode 131 si celui-ci est indiqué. L’association d’une chirurgie thyroïdienne macroscopiquement complète, d’une irathérapie et de l’administration de L-thyroxine permet de contrôler la maladie dans la quasi-totalité des cas. Ainsi, cette trithérapie maximaliste assure un bon pronostic de ces formes évoluées et pourrait augmenter le taux de rémission complète [18,19]. L’extension peut se faire vers les poumons qui reste la localisation secondaire la plus fréquente et qui prennent l’aspect d’une miliaire isotopique au balayage du corps entier, rarement révélées sur une radiographie standard des poumons, et dont l’évolution est généralement très satisfaisante comme en témoigne le taux de thyroglobuline effondré chez notre patient avec miliaire isotopique [20]. La récidive ganglionnaire est important, évalué de 21 à 29%, mais qui reste curable, grâce à l’association chirurgie et d’iode radioactif [9, 21]. Le pronostic est globalement favorable même en cas d’extension initiale importante et même après rechute locale, avec une survie à 20 ans supérieure à 90%. Une étude récente portant sur 215 enfants a rapporté une mortalité spécifique à 40 ans de 2% [14]. Le suivi après traitement a deux objectifs: d’une part, adapter l’hormonothérapie, et d’autre part, dépister précocement les rechutes. Après le traitement initial, un traitement par L-thyroxine est institué. Chez les enfants dont la scintigraphie post thérapeutique initiale est normal, un bilan est pratiqué neuf à douze mois plus tard, et comprend un dosage de la thyroglobuline après stimulation par la TSH et une échographie cervicale [22]. La pratique d’autres examens, notamment d’un examen scintigraphique du corps entier à l’iode 131 n’est indiquée qu’en cas d’anomalies sur ces examens. Par la suite, la surveillance est annuelle.

## Conclusion

Le CDT est une maladie rare chez l’enfant et l’adolescent, le type histologique papillaire reste le plus fréquent. En dépit de la nature agressive de ces cancers chez l’enfant par rapport aux adultes, la survie globale reste excellente après traitement, qui associe une thyroïdectomie totale avec au minimum un curage central, complété par une irathérapie. Sa surveillance est basée sur le dosage de la thyroglobuline et doit être poursuivie toute la vie en raison de la possibilité de rechutes très tardives.

### Etat des connaissances actuelle sur le sujet

- Le cancer différencié de la thyroïde de l’enfant et l’adolescent est une entité rare mais agressive;
- Le type histologique papillaire reste le plus fréquent;
- La survie globale reste excellente après traitement.

### Contribution de notre étude à la connaissance

- C’est une entité rare;
- C’est la plus grande série au Maroc;
- C’est une série de cas pour exploitation internationale.

## Conflits d’intérêts

Les auteurs ne déclarent aucun conflit d'intérêt.
